# Extending pathways and processes using molecular interaction networks to analyse cancer genome data

**DOI:** 10.1186/1471-2105-11-597

**Published:** 2010-12-13

**Authors:** Enrico Glaab, Anaïs Baudot, Natalio Krasnogor, Alfonso Valencia

**Affiliations:** 1 School of Computer Science, Nottingham University, Jubilee Campus, NG81BB Nottingham, UK; 2 Structural Biology and Biocomputing Program, Spanish National Cancer Research Centre, CNIO, E-28029 Madrid, Spain; 3 Luminy Institute of Mathematics, UMR6206, Campus de Luminy, Case 907, 13288 Marseilles Cedex 9, France

## Abstract

**Background:**

Cellular processes and pathways, whose deregulation may contribute to the development of cancers, are often represented as cascades of proteins transmitting a signal from the cell surface to the nucleus. However, recent functional genomic experiments have identified thousands of interactions for the signalling canonical proteins, challenging the traditional view of pathways as independent functional entities. Combining information from pathway databases and interaction networks obtained from functional genomic experiments is therefore a promising strategy to obtain more robust pathway and process representations, facilitating the study of cancer-related pathways.

**Results:**

We present a methodology for extending pre-defined protein sets representing cellular pathways and processes by mapping them onto a protein-protein interaction network, and extending them to include densely interconnected interaction partners. The added proteins display distinctive network topological features and molecular function annotations, and can be proposed as putative new components, and/or as regulators of the communication between the different cellular processes. Finally, these extended pathways and processes are used to analyse their enrichment in pancreatic mutated genes. Significant associations between mutated genes and certain processes are identified, enabling an analysis of the influence of previously non-annotated cancer mutated genes.

**Conclusions:**

The proposed method for extending cellular pathways helps to explain the functions of cancer mutated genes by exploiting the synergies of canonical knowledge and large-scale interaction data.

## Background

Processes and pathways, whose deregulation may contribute to the development of cancers [1], are often represented as cascades of proteins transmitting a signal from the cell surface to the nucleus. However, the delineation of the canonical members of these cellular pathways is based on a multitude of experimental methods, and some inconsistencies exist across databases [2]. Indeed, the assignment of a protein to a pathway often relies on the experimental procedure and on a subjective assessment of the protein's importance for the process. Many closely associated regulators, effectors or targets of cellular pathways may therefore have been overlooked by these classical approaches. Additionally, recent functional genomics high-throughput initiatives have identified a large number of interaction partners for signalling proteins, suggesting more complex relationships between cellular pathways than in their traditional representations [3]. In this context, the analysis of cancer mutated genes at the level of canonical cellular processes and pathways may previously have missed potentially interesting findings.

This paper introduces a new methodology to amalgamate the information from cellular process and pathway databases with large-scale protein-protein interaction data. Previous approaches for in-silico generation of cellular processes based on molecular interaction data have constructed pathways from scratch (see [4-7]), and related approaches for disease candidate gene prioritisation also rely on interaction network data to identify molecules which are associated with a gene set [8-10]. However, to the best of the authors' knowledge an extension approach which preserves existing process definitions has not yet been investigated.

Here, we present a procedure for extending cellular pathways and processes by mapping them onto a protein-protein interaction network and identifying densely interconnected interaction partners. Briefly, we map proteins annotated for different cellular processes onto a large protein-protein interaction network, and extend each of these processes by adding their most densely interconnected network partners (using various graph-theoretic criteria). These added proteins display distinctive network topological features and molecular function annotations and can be proposed as putative new components of the corresponding cellular process, and/or as regulators of the communication between different cellular processes. This is illustrated by the prediction of new Alzheimer disease candidate genes and the identification of proteins with potential involvement in the crosstalk between several interleukin signalling pathways.

Finally, we employ the extension procedure to investigate mutated genes from a large-scale resequencing study of pancreatic tumours. We identified many pathways and processes enriched in mutated genes, as well as cancer mutated genes predicted to be involved in specific pathway deregulations.

## Implementation

All data processing and analysis steps were implemented in the programming language R. The web-based pathway visualisation on http://www.infobiotics.net/pathexpand was implemented in PHP.

### Interaction network construction

The human protein-protein interactions were combined from 5 public databases, as of July, 2009. These include MIPS [11], DIP [12], MINT [13], HPRD [14] and IntAct [15]. We considered only experimental methods dedicated to the identification of direct binary protein interactions (see datasets section on the webpage http://www.infobiotics.net/pathexpand). The final protein interaction network contained 9392 proteins (nodes) and 38857 interactions (edges).

### Process mapping

The gene/protein sets corresponding to cellular pathways/processes were extracted from the public databases KEGG [16], BioCarta [17] and Reactome [18] and were mapped onto the protein interaction network. Since the interaction data does not represent the entire proteome, on average about 60% of the pathway proteins could be mapped onto the network.

### Process extension procedure

**Figure 1:**
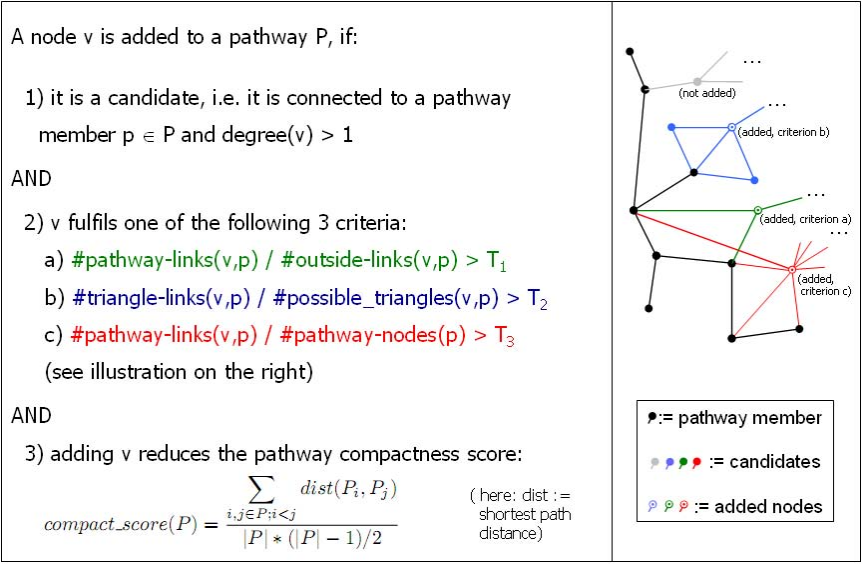
**Filtering criteria**. Visualisation of graph-based filtering criteria used to extend the cellular processes (the process nodes are shown in black, coloured and circled nodes represent cases in which different filtering criteria are fulfilled by a candidate node).

Original cellular pathways/processes containing at least 10 proteins were used as seeds and mapped onto the protein-protein interaction network. The direct neighbours of these seed nodes were then considered as candidates for the extension procedure, and filtered according to multiple criteria to assess the strength of their association with the pathway nodes. More specifically, in the first filtering step, a candidate-node v has to fulfill condition (1) below and at least one of the following conditions (2-4) to be added to a pathway p (an illustration of these conditions is shown in Figure 1).

**node degree**:

(1) degree(v)>1

**direct pathway/process association**:

(2) $$\frac{process\text{\_}links(v,p)}{outside\text{\_}links(v,p)}>T_{1}$$

**Process extension procedure**:

(3) $$\frac{triangle\text{\_}links(v,p)}{possible\text{\_}triangles(v,p)}>T_{2}$$

pathway/process node coverage:

(4) $$\frac{process\text{\_}links(v,p)}{process\text{\_}nodes(p)}>T_{3}$$

where *degree*(*v*) is the number of direct links of node *v*, *process_links*(*v*, *p*) is the number of direct links from *v *to a node in the process *p *and *outside_links*(*v, p*) is the number of direct links from *v *to a node outside of process *p*. *triangle_links*(*v, p*) is the number of triangles in which *v *occurs together with a node in *p *and another candidate-node, and *possible_triangles*(*v*, *p*) is the number of these triangles which could potentially be formed, if all other candidate nodes would be part of a triangle formed together with *v *and *p*. *T*_1_, *T*_2 _and *T*_3 _are defined here as *T*_1 _= 1.0, *T*_2 _= 0.1 and *T*_3 _= 0.3 (this selection provided a reasonable trade-off between the number of extended pathways and the average size of the extension). For *T*_1 _= 1.0, equation 2 corresponds to a well-known condition in graph theory introduced to define "strong communities" in networks (stating that the number of connections to the pathway/community must exceed the number of connections to the rest of the graph, see [19]). Given that a candidate node can have connections with all the original pathway nodes, the threshold *T*_3 _always has to be smaller than 1 (i.e. the maximum pathway node coverage is 1).

Since we expected the extension procedure to be more meaningful if it results in a more compact pathway-representation in the network, we apply a second filter to the candidate nodes which passed the first fillter based on the above criteria. A candidate node is only accepted if the following compactness-score, given by the mean of the shortest path lengths between all pairs of proteins belonging to a protein set P, is reduced after adding the candidate:

(5) $$compact\text{\_}score(P)=\frac{\sum_{i,j\in P;i<j}dist(P_{i},P_{j})}{|P|*(|P|-1)/2}$$

Thus, the proteins that are added to a pathway by the procedure are both strongly associated with the original pathway members and provide an extended pathway with a compact network representation. Specifically, we expect that added proteins which increase the compactness by connecting disconnected proteins in the original pathway are more likely to be functionally similar to the pathway members. The order in which proteins are added to extend a pathway is given by a greedy strategy, i.e. the protein that increases the compactness the most is always added first.

### Topological network analysis

To quantify local and global topological properties of proteins in the network, we used the web-application TopoGSA [20] to compute five topological descriptors: the number of connections to other nodes (degree), the tendency of nodes to form clusters (clustering coefficient), their centrality in the network (betweenness and eigenvector centrality) and the distances between them (shortest path length). For a detailed explanation of these topological characteristics, see [21].

### Cross-validation

We applied the following cross-validation strategy to analyse the extent to which randomly deleted proteins in the original pathways/processes can be recovered by our extension procedure:

1. 10% of the proteins from each pathway were removed randomly among those proteins that are connected to at least one other protein in the pathway. If the set of proteins that are connected to other pathway members covers less than 10% of the total number of proteins, we iteratively remove random proteins from this set and recompute the set until it is empty.

2. To each reduced pathway the proposed extension procedure was applied as well as 100 alternative random extensions, computed by sampling randomly the same number of proteins from the *candidate *proteins of the reduced pathway (see definition of *candidates *in the process extension section above).

3. P-value significance scores are estimated as the relative frequency of cases where more proteins were correctly recovered by a random extension than by the proposed extension procedure across all pathways in a database.

### Semantic similarity analysis of Gene Ontology terms

We quantified pairwise similarities between protein annotations based on Jiang and Conrath's semantic similarity measure for GO terms [22]. Using this similarity score, we computed the average GO-term similarities between all pairwise combinations of GO biological process (BP) terms for the original proteins in the cellular pathway and the added proteins. A random extension model was created by randomly sampling the same number of proteins from the candidate proteins of the pathway (see definition of candidates in the pathway extension section) as in the real extension, excluding the proteins from the extended cellular pathway under consideration. The reader should note that it is not possible to compare the extensions of real pathways to extensions of random gene/protein sets with similar connectivity in the network, because in most cases these sets would largely overlap with other pathways.

### Enrichment Analyses

• The enrichment of molecular functions among the proteins added to the cellular pathways/processes by the extension procedure was tested for all databases independently using the DAVID functional annotation clustering tool [23] (Gene Ontology Molecular Functions and InterPro protein domains), with the proteins from the interaction network. Functional annotation clusters with a more than 2-fold enrichment were selected and manually labelled.

• To estimate the probability of observing certain overlaps between extended or original protein sets representing pathways and other protein sets of interest, e.g. cancer-related proteins, we used a classical over-representation analysis (ORA) based on the one-tailed Fisher exact test. To adjust for multiple testing, we employ the approach by Benjamini [24].

## Results and Discussion

In the following we discuss the results obtained by applying our pathway extension approach to cellular pathway and process datasets from the databases KEGG [16], BioCarta [17] and Reactome [18]. Across all databases, 1859 different processes were considered (with a minimum size of 10 proteins) and mapped onto a network containing 38857 interactions (see Methods).

### Extension of cellular pathways/processes with protein interaction data predicts new putative components

Our procedure has been able to extend 159 pathways from BioCarta, 90 from KEGG and 52 from Reactome (Table 1http://www.infobiotics.net/pathexpand). The pathway sizes increased on average from 113% to 126% of the original size.

**Table 1:** Statistics on added proteins across different databases

Property	BioCarta	KEGG	Reactome
no. of examined pathways	322	199	79

no. of extended pathways	195	140	62

avg. pathway size	19	49	75

avg. size after extension	24	61	85

total no. of added proteins	935	1745	622

no. of unique added proteins	280	623	409

Molecular function categories of proteins added by the extension method (2-fold enrichment, see methods)	Phosphatase activity, Regulator activity, Binding, Kinase inhibitor/regulator, Cytokine binding/TNF receptor	Phosphatase activity, Regulator activity, Cytokine binding/TNF receptor	Regulator activity

Statistics on the number of pathways that could be extended, the average extension size, the number of added (unique) proteins and their molecular function categories.

### Network properties of the proteins added to the cellular pathways/processes

The added proteins in the interaction network had a more than one standard deviation higher node degree, betweenness and average local clustering coefficient (Methods) than 10 matched size random protein sets [20] (Table 2). Moreover, the shortest path lengths between the added proteins were smaller by several standard deviations (Table 2). This tendency of proteins added by the extension method to occur in more central and dense regions of the network is consistent with similar trends observed for the topological properties of proteins from the original cellular pathways and processes (Table 2).

**Table 2:** Topological properties of BioCarta pathway/process extensions 17

Property	Proposed extension: Added proteins only (mean)	Random model: Added proteins only (mean/stddev.)	Original cellular processes (mean/stddev.)	All network proteins (mean/stddev.)
Shortest path length	3.68	4.11(0.03)	3.77 (0.51)	4.12(0.94)

Node betweenness	21998	14545(4751)	49888 (153173)	14669(68893)

Degree	10.3	8.11(0.94)	21.53 (32.64)	8.27(16.2)

Clustering coefficient	0.34	0.11(0.01)	0.12 (0.17)	0.11(0.21)

Eigenvector centrality	0.04	0.01(0.04)	0.05 (0.09)	0(0.57)

Comparison of different numerical topological properties for the proteins added by the proposed extension method (column 1) or the random model (column 2), as well as a comparison of these properties for the nodes corresponding to the original cellular processes (column 3) and the entire protein-protein interaction network (column 4).

### Functional annotations of the proteins added to the cellular pathways/processes

**Figure 2:**
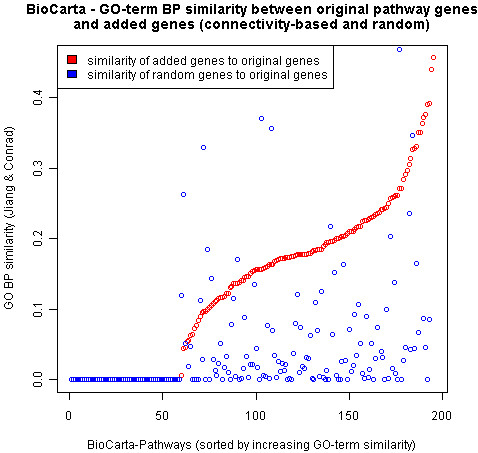
**Semantic similarity analysis**. Similarities in Gene Ontology Biological Process terms between original BioCarta pathway proteins and added proteins (red) and between original pathway proteins and matched-size random protein sets (blue).

A semantic similarity analysis of the GO terms was used to compare the functional annotations of the original cellular process proteins with the annotations of the proteins added during the extension procedure (Methods). For almost all cellular pathways, the GO-terms of the added proteins are more similar to the GO-terms of the original cellular pathway proteins than those of matched-size random protein sets (Figure 2). These results confirm that the added proteins belong to similar functional categories as the proteins from the cellular processes they were assigned to. Furthermore, a functional enrichment analysis of the combined set of proteins added to all cellular processes (applied to each database separately) reveals an enrichment in proteins annotated for regulatory activity (Table 1). More interestingly, for the databases KEGG and BioCarta, the added proteins are enriched in phosphatases. This result could indicate that phosphatases, which might correspond to negative regulators, have previously been overlooked in the definition of canonical pathways.

### The extension procedure can recover known pathway proteins after deletion

A cross-validation procedure (Methods) showed that the cellular pathway extension recovers a Significantly larger number of randomly deleted pathway- nodes in the network than a simplistic extension based on random selection among the candidate nodes (p-values smaller than 0.01 for all databases). Specifically, the distribution of the number of recoveries across the 100 random model extensions never provided a higher number of recoveries than the proposed extension method.

### Prediction of new components

Based on the observations that 1) The proteins added by our method are well connected and central in the protein interaction network, 2) The added proteins display gene ontology annotations matching better to the original cellular pathway/process annotations than random proteins, and are enriched in processes known to be related to cellular signalling, and 3) Our method is able to recover known cellular pathway/process proteins in a cross-validation experiment, we propose to consider the proteins added by the extension procedure as new candidate components with a functional role in the corresponding cellular processes.

To illustrate the utility of our extension procedure for the prediction of new components, we analysed a cellular map modelling the process likely to be deregulated by the most penetrant Alzheimer susceptibility genes (created manually from the literature [25] and available in the KEGG database [16]). Our extension method added 5 different proteins to this cellular map http://www.infobiotics.net/pathexpand. Interestingly, three of them have previously been implied in Alzheimer disease (TMED10, APH1B and PITX3). Two other proteins, METTL2B and MMP17, which are also added to the Alzheimer cellular map by our method, have not been linked to the disease so far, to the best of our knowledge. MMP17 is a member of the metallopeptidase protein family involved in the breakdown of the extracellular matrix. According to the Huge navigator [26], 6 other members of this protein family have been associated with the Alzheimer disease. The other candidate is a methyltransferase-like, METTL2B. Another member of this family, MMETL10 has been associated with Alzheimer disease in a case-control study [26]. Thus, using the Alzheimer disease pathway as a first test case of our method, we can propose MMP17 and METTL2B as new candidate disease genes.

### The extension of cellular processes points to extensive communication

The involvement of some proteins in multiple processes suggests that extensive communication exists between different cellular processes. Indeed, before applying the extension procedure, about 50% of the cellular process proteins are annotated for more than one cellular process. Interestingly, after the extension procedure, the percentage of unique proteins among all proteins added to the cellular processes ranged from 30% (BioCarta) to 66% (Reactome), revealing that many proteins are added to more than one cellular process. In agreement with our observations for the original process proteins, again about 50% of the added proteins belong to more than one cellular process. Accordingly, many proteins in the protein interaction network are well connected with different cellular processes, and might therefore be expected to have a functional role in the communication between the cellular processes.

**Figure 3:**
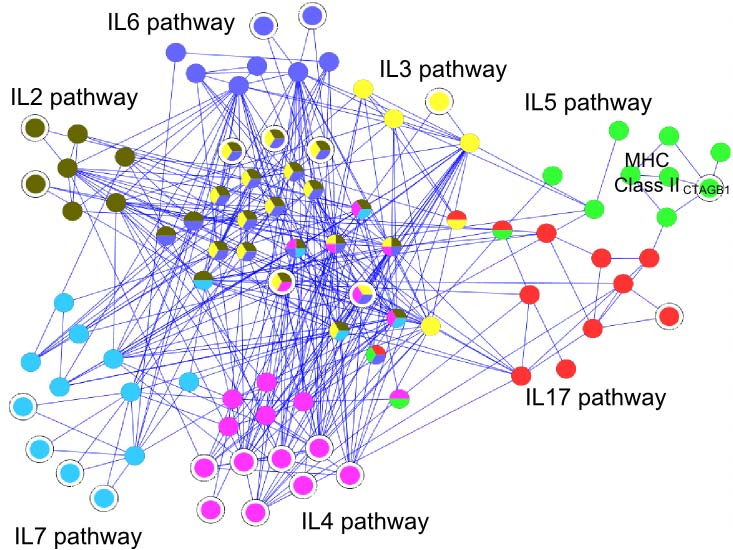
**Crosstalk between interleukin signalling pathways**. Protein interaction sub-network containing the proteins annotated for 7 different Interleukin (IL)-related pathways from the BioCarta database (each colour represent a pathway, proteins annotated for multiple pathways display more than one colour). Proteins added by our method are highlighted by surrounding circles and coloured according to the pathway(s) they were added to (they appear mostly within peripheral clusters or as links between process members). They were not annotated for any of the IL-related pathways before applying the extension procedure, and the original pathway members did not become members in further IL-related pathways. Therefore, to simplify interpretation and provide a compact data representation, the node colours are only used to visualise the pathway memberships after the application of the extension procedure.

As an example for these type of connections, we consider the class of Interleukins (ILs). ILs are secreted proteins mainly involved in the immune system to regulate the communication between immune cells. They activate different signalling pathways, which can share intracellular signalling cascades (e.g., MAPK, Ras or STAT), but which also display distinct properties (e.g. by binding to different receptors). In this context, some IL-pathway proteins are annotated only for one IL pathway (Figure 3 each colour corresponds to an IL pathway), while other proteins occur in multiple pathways (Figure 3 multiple colour node proteins). Furthermore, all the IL pathways share protein-protein interactions (Figure 3 blue links). Thus, the analysis of protein-protein interactions between the members of different IL pathways highlights the complexity of this signalling system. We applied our pathway extension method to extend the seven interleukin signalling pathways depicted in Figure 3 (between 1 to 10 proteins were added to each signalling pathway). The figure shows that some proteins were added to only a single IL pathway. For instance, the CTAG1B (cancer/testis antigen 1B) protein was added to the IL5- signalling pathway (Figure 3 green proteins). Interestingly, the added protein is an antigen expressed only in cancer cells and in normal testis cells, and could represent a regulatory member of this pathway in these two particular conditions. Moreover, four other proteins were added jointly to more than one IL pathway. Three of them are added to the IL2, IL3 and IL6 pathways, which are all activating the STAT and Ras/MAPK signalling cascades. These proteins are known regulators of these cascades and can also participate in the regulation of the communication between the different interleukin signalling pathways.

### Functional enrichment of tumour mutated genes in extended cellular pathways reveals new putative regulators of cancer pathways

Large-scale tumour resequencing projects have revealed a large number of genes mutated in different cancer types [27-29]. To understand the biological significance of these mutated genes, those cellular processes containing more mutated genes than expected by chance have been identified (see for instance [28]).

We applied an enrichment analysis on cancer mutated genes extracted from a pancreatic large-scale resequencing study [28], with extended cellular processes from BioCarta, KEGG and Reactome, and identified significant associations between different cancer types and the extended pathways (Methods).

Interestingly, we retrieve 8/12 core signalling pathways that have previously been identified as significantly associated with this disease [28]. An over-representation analysis (ORA) shows that some cellular pathways and processes are more significantly enriched in mutated genes in the extended versions than in the original versions (Table 3). These include signalling pathways, such as MAPK, p38 MAPK, p53, Wnt, PDGF, FC epsilon receptor I, ErbB or functions such as apoptosis and cell cycle G1/S check point (Table 3). Interestingly, some of the proteins added to these processes during the extension procedure are also pancreatic mutated genes (Table 3). These proteins include, for instance, the BCL2-related protein A1, which is added by our method to the Apoptosis Reactome pathway (indeed, this protein is annotated as being involved in apoptosis). A less obvious example is the dual specificity phosphatase 19 (DUSP19), a phosphatase added by the extension procedure to MAPK pathways, the Fc epsilon receptor I signalling pathway and to a pathway known to be activated in response to HIV Nef protein (negative effector of Fas and TNF). This protein is highly expressed in the pancreas [30] and displays a frameshift mutation in pancreatic tumours [28].

**Table 3:** Cellular processes enriched in pancreatic mutated genes

Cellular Process database	Cellular process	ORA Q-value before/after extension	Pathway size before/after extension	Number of mutated genes in new pathway	Number of mutated genes among added genes	Mutated genes among added genes
Reactome	Hemostasis	0.475/5.18e-06	221/278	19	4	LRP1B, TFPI2 PON1, SIGLEC11

KEGG	Tight junction	1.48E-4/4.5e-05	106/126	14	3	RASIP1, RASGRP3, PLEKHG2

KEGG	MAPK signaling pathway	3.35E-4/4.87e-05	225/279	21	6	DOCK2, MAPKBP1, SLC9A5 RASIP1, DUSP19, PLEKHG2

KEGG	Cell adhesion molecules	2.87E-4/1.03E-4	109/116	12	2	TNR, SEC14L3

KEGG	Wnt signaling pathway	3.35E-4/1.39E-4	123/147	14	3	MAPKBP1, PLEKHG2, ANKRD6

KEGG	Neuroactive ligand- receptor interaction	3.35E-4/1.72E-4	198/217	17	3	EML1, ACE

BioCarta	MAPKinase Signaling Pathway	1.33E-3/2.89E-4	81/111	8	2	MAPKBP1, DUSP19

Reactome	Apoptosis	3.7E-2/4.42E-4	124/146	11	2	BCL2A1, RASGRP3

Reactome	Signaling by PDGF	5.72E-3/4.43E-4	61/121	10	3	VPS13A, LIG3 FMR2

BioCarta	Cell Cycle G1/S Check Point	1.7E-3/5.06E-4	27/34	5	1	TGIF2

BioCarta	Agrin Postsynaptic Differentiation	1.27E-2/8.21E-4	27/38	5	2	PGM5, PLEKHG2

BioCarta	p38 MAPK Signaling Pathway	3.25E-3/1.13E-3	34/42	5	1	PLEKHG2

BioCarta	ALK in cardiac myocytes	2.89E-3/1.25E-3	32/44	5	1	TBX5

KEGG	Fc epsilon RI signaling pathway	2.69E-2/2.71E-3	67/114	10	5	DOCK2, MAPKBP1, DUSP19, ATF2, RASGRP3

KEGG	ErbB signaling pathway	2.32E-2/3.52E-3	86/196	13	7	VPS13A, MAPKBP1, NEK8, LIG3, DUSP19, AFF2, GLTSCR1

KEGG	Regulation of actin cytoskeleton	4.94E-3/2.72E-3	184/236	15	4	RASIP1, CDC42BPA, PLEKHG2, CYFIP1

BioCarta	HIV-I Nef negative effector of Fas and TNF	7.88E-3/4.78E-3	50/66	5	1	DUSP19

KEGG	p53 signaling pathway	5.62E-3/5.44E-3	59/64	7	1	PPP2R4

Reactome	Signaling in Immune system	0.459/7.02E-3	228/266	12	1	SEC14L3

The complete list of cellular processes that display a statistically significant enrichment in pancreatic cancer mutated genes after applying the proposed extension method (Q-value < 0.01) and improved significance scores in relation to the original pathways (i.e. Q-values decreasing after the extension). The significance scores for the overrepresentation analysis (ORA) and the pathway sizes are shown before and after the extension, and the total number of mutated genes in the extended pathways is provided, as well as the size and the annotations for the set of mutated genes among the genes that were added to these pathways.

**Figure 4:**
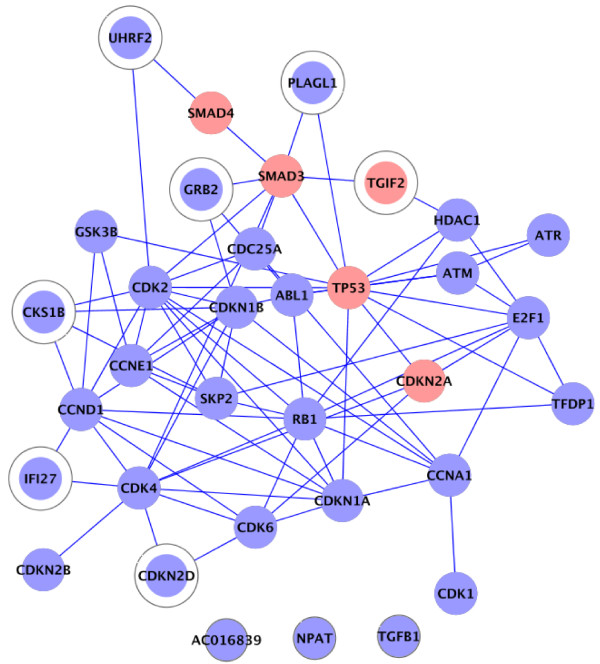
**Cell cycle G1/S check point subnetwork**. Protein-protein interaction subnetwork corresponding to the proteins annotated for the BioCarta pathway "Cell cycle G1/S check point" and proteins added by our extension procedure (circled). Proteins whose corresponding genes have been found mutated in pancreatic whole-genome resequencing studies [28] are highlighted in red.

Finally, new insights can be gained when analysing the BioCarta cell cycle G1/S check point process (Figure 4). This process contains several proteins that were found mutated in large-scale pancreatic resequencing studies (Figure 4 red nodes), as well as many other proteins known to be involved in cancerogenesis. Our extension procedure adds seven proteins to this process (Figure 4 circled nodes). All of these proteins are either transcription factors, kinases or other signal transduction regulators, and six of them are known to be involved in cell cycle regulation (all except TGIF2), though not belonging to the BioCarta canonical cell cycle G1/S check point proteins. Interestingly, the cancer resequencing study showed the TGIF2 gene to be mutated in a pancreatic tumour (Figure 4 circled red node). This transcriptional repressor gene has also been reported to be amplified in some ovarian cancers, and can be recruited by TGF-β-activated Smads [31]. We predict both the involvement of the corresponding TGIF2 protein in the cell cycle G1/S check point process, and its involvement in cancer through the deregulation of this process.

In conclusion, the extensions of the cell cycle G1/S and other processes provide useful explanatory information for the cancer association of these pathways/processes by adding new regulators that increase the connectivity between cancer mutated genes and other process members in the interaction network. For instance, in the G1/S process, SMAD3 is connected to other process members by adding the proteins TGIF2, GRB2 and PLAGL1, and SMAD4 is connected to the process member CDK2 by adding UHRF2. Thus, the overall coherence of the processes is increased and an expanded view of the influence of different cancer genes in these processes is obtained.

## Conclusions

The extension of known cellular pathways and processes with densely interconnected interaction partners in a protein-protein interaction network leads to the proposal of new putative components and to the identification of mediators of the communication between the processes. Thus, by taking into account canonical knowledge as well as large-scale interaction data, the extended pathways help to explain the functions of cancer mutated genes.

## Availability and requirements

The web-based pathway visualisation, details about the generation of the human protein-protein interaction network and the complete enrichment analysis results are freely available at http://www.infobiotics.net/pathexpand.

## Authors' contributions

AB and EG jointly drafted the manuscript. EG participated in the design of the study, implemented the algorithm and web-based visualisation, and carried out the statistical analyses. AB participated in the design of the study, collected the cancer mutated gene, cellular pathway and interaction data, and analysed the functional annotations of added proteins and the enrichment results. AV and NK took part in the design and coordination of the study, provided input into main ideas of the paper and obtained funding for the project. All authors read, commented and approved the final version of the manuscript.
